# Context-specific effects of facial dominance and trustworthiness on hypothetical leadership decisions

**DOI:** 10.1371/journal.pone.0214261

**Published:** 2019-07-29

**Authors:** Hannah S. Ferguson, Anya Owen, Amanda C. Hahn, Jaimie Torrance, Lisa M. DeBruine, Benedict C. Jones

**Affiliations:** 1 Department of Psychology, Humboldt State University, Arcata, CA, United States of America; 2 Institute of Neuroscience and Psychology, University of Glasgow, Glasgow, United Kingdom; Leiden University, NETHERLANDS

## Abstract

Social judgments of faces predict important social outcomes, including leadership decisions. Previous work suggests that facial cues associated with perceptions of dominance and trustworthiness have context-specific effects on leadership decisions. Facial cues linked to perceived dominance have been found to be preferred in leaders for hypothetical wartime contexts and facial cues linked to perceived trustworthiness have been found to be preferred in leaders for hypothetical peacetime contexts. Here we sought to replicate these effects using images of women’s faces, as previous studies have primarily focused on perceptions of leadership abilities from male faces, with only a handful of these including female faces. Consistent with previous work, a linear mixed effects model demonstrated that more trustworthy-looking faces were preferred in leaders during times of peace and more dominant-looking faces were preferred in leaders during times of war. These results provide converging evidence for context-specific effects of facial cues on hypothetical leadership judgments.

## Introduction

Social judgments of faces predict important social outcomes, such as romantic and platonic partner choices and hiring decisions (e.g., [1,2]). One area that has received considerable attention in the social perception literature is the role that social judgments of faces play in hypothetical leadership decisions (reviewed in [3]). Indeed, several lines of evidence suggest that even very rapid social judgments of politicians’ faces predict actual election outcomes [4–6].

Several studies have found that people whose faces are judged to look particularly dominant or trustworthy are preferred in hypothetical leadership decisions [7]. However, other research suggests that facial characteristics linked to perceptions of dominance and trustworthiness can have context-specific effects on hypothetical leadership decisions [3,7]. People with masculine faces are generally perceived to look more dominant [8] and tend to be preferred as leaders in hypothetical wartime scenarios [9]. By contrast, people with feminine faces are generally perceived to look more trustworthy [8] and tend to be preferred as leaders in hypothetical peacetime scenarios [9,10]. These results have been interpreted as evidence that stereotypic perceptions of candidates’ suitability for particular types of leadership roles influence hypothetical leadership decisions, potentially reflecting the context-specific relevance of these traits for different types of coalitions [3,4,10].

Spisak et al. [9] demonstrated context-specific effects of facial appearance on leadership decisions using face stimuli that had been experimentally manipulated along a masculinity-femininity dimension. Some researchers have criticized this method (i.e., experimental manipulation of facial characteristics) because the observed effects on perceptions may not generalize well to judgments of natural face images that vary simultaneously on many dimensions (e.g., [11]). Because of such criticisms, we attempted to conceptually replicate Spisak et al’s results for context-specific leadership judgments using dominance and trustworthiness ratings of unmanipulated face images. Unmanipulated female faces, varying naturally in perceived dominance and trustworthiness, were judged for leadership ability in a hypothetical peacetime or wartime scenario. Given Spisak et al’s [9] findings, we predicted that more dominant-looking faces would be preferred in leaders during the wartime context, while more trustworthy-looking faces would be preferred in leaders during the peacetime context.

## Methods

### Stimuli

Standardized full-color photographs of 50 young adult white women (mean age = 24.3 years, SD = 4.01 years) were used in this study. The women posed front-on to the camera with direct gaze and neutral expressions to control for possible effects of gaze and emotion cues on responses to faces. Images were aligned on pupil position and cropped so that clothing was not visible. Faces from this image set have been used in several previous studies of social judgments from facial cues (e.g., [12–14]).

### Dominance/Trustworthiness ratings

One hundred heterosexual men (mean age = 26.22 years, SD = 6.11 years) and 100 heterosexual women (mean age = 24.71 years, SD = 5.4 years) were recruited online (from social bookmarking websites, such as stumbleupon.com) between October 2010 and March 2011. Raters provided written consent and the researchers did not have access to any identifying rater information. These raters were randomly allocated to rate the female face images for either trustworthiness or dominance using 1 (very low) to 7 (very high) scales. There were no time restrictions on these ratings, and all ratings were collected using in-house software. Inter-rater agreement was high for both sets of ratings (both Cronbach’s alphas > .90).

### Perceived leadership ability

A different group of 137 men (mean age = 29.4 years, SD = 10.91 years) and 237 women (mean age = 25.45 years, SD = 9.43 years) were recruited online between October 2016 and May 2017, with no exclusion criteria applied. Raters provided written consent and the researchers did not have access to any identifying rater information. Using a between-subject design, these raters were randomly allocated to rate the same 50 female faces for leadership at a time of war *or* leadership at a time of peace, also using a 1 (very bad) to 7 (very good) scale. The specific questions asked for leadership ratings were “How good a leader would this person be for a country during a time of war?” and “How good a leader would this person be for a country during a time of peace?”. Although less ecologically valid than an actual voting task, this type of hypothetical leadership judgment has been used in a number of previous studies [4,7,9,10] and social judgments of faces have been linked to actual election outcomes [6]. Trial order was fully randomized for all ratings. There were no time restrictions on these ratings, and all ratings were collected using in-house software.

## Results

Data were analyzed using a linear mixed effects model with leadership rating as the dependent variable and average dominance rating, average trust rating, and leadership context as predictors. The model also included interactions between dominance rating and leadership context and between trustworthiness rating and leadership context. Random intercepts were specified for the 50 faces and the 373 participants. Random slopes were specified maximally (dominance and trustworthiness ratings by participant and context by face). Data and analysis code are publicly available at https://osf.io/q54nm/.

**Fig 1:**
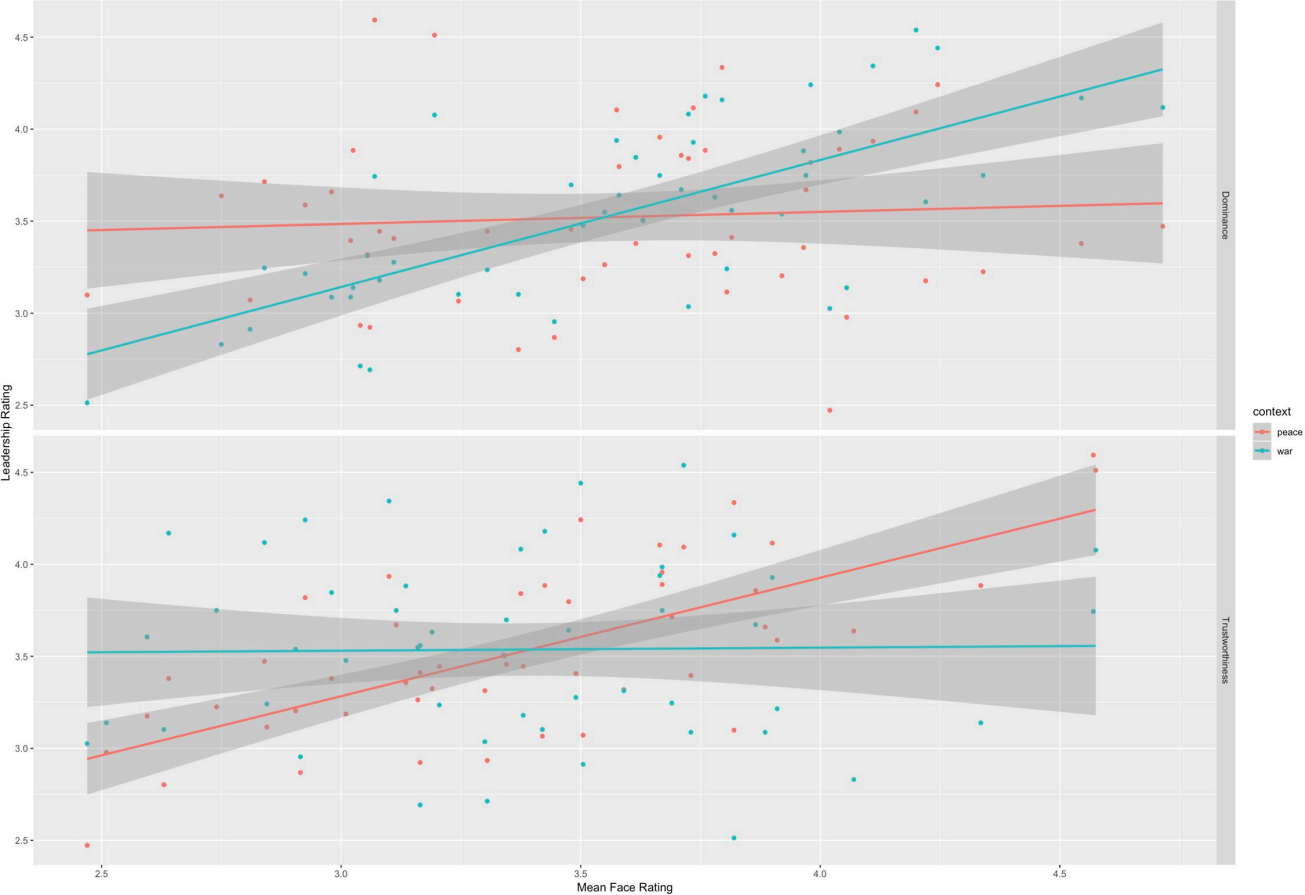
Context-specific effects of facial dominance and trustworthiness on leadership judgments. Relationship between perceived leadership ability and facial dominance (top) and trustworthiness (bottom) during times of peace (blue) and war (red).

The relationship between leadership ratings and dominance ratings was qualified by context (beta = 0.38, SE = 0.08, t(149.99) = 4.59, p < .001, 95% CI = [0.22, 0.55]). The relationship between leadership ratings and trustworthiness ratings was also qualified by context (beta = -0.4, SE = 0.08, t(129.37) = -4.93, p < .001, 95% CI = [-0.55, -0.24]). As predicted, dominance ratings were positively related to leadership ratings in the war, but not peace, context, while trustworthiness ratings were positively related to leadership ratings in the peace, but not war, context (see Fig 1).

## Discussion

The current study investigated hypothetical leadership decisions using female faces that varied naturally in perceived dominance and trustworthiness for two different leadership contexts; peacetime and wartime. Consistent with Spisak et al’s [9] findings, our results indicate that women with more dominant-looking faces were preferred as leaders during hypothetical wartime contexts, while women with more trustworthy-looking faces were preferred as leaders during hypothetical peacetime contexts.

While previous research has focused primarily on leadership decisions of male faces, our results add to a growing body of research demonstrating that the effects of facial appearance on perceived leadership ability extend to female faces [15,16]. Although some previous research has suggested that manipulating the masculinity of female faces does not increase perceived dominance as much as it does in male face counterparts [16,17], several studies investigating the impact of facial cues on leadership decisions have demonstrated that cues of masculinity-femininity are more influential than actual sex cues in determining leadership decisions in hypothetical voting tasks [9,18]

The pattern of results that we observed provides converging evidence for the hypothesis that facial cues have context-dependent effects on leadership decisions. In their evolutionary-contingent hypothesis of these context-specific judgments of leadership abilities, van Vugt and Grabo [3] argue that these judgments may reflect attunement to the ability of individuals to solve specific intergroup challenges in human history. Although evidence that perceptions of other peoples’ dominance and trustworthiness are accurate is mixed and controversial [2], our findings present further evidence that stereotypic perceptions of faces shape social judgments in ways that are predictable and somewhat rational.
